# Prevalence of hepatitis B surface antigen (HBsAg) and its risk factors among individuals visiting Goba General Hospital, South East Ethiopia, 2012

**DOI:** 10.1186/1756-0500-7-833

**Published:** 2014-11-24

**Authors:** Asfaw Negero Erena, Tomas Benti Tefera

**Affiliations:** Department of Medicine, Madawalabu University, College of Medicine and Health Sciences, Bale-Goba, South East Ethiopia; Department of Nursing, Madawalabu University, College of Medicine and Health Sciences, Bale-Goba, South East Ethiopia

**Keywords:** Hepatitis B Virus (HBV), Human Immuno Deficiency Virus (HIV), Co infection, Madawalabu University, Goba Ethiopia

## Abstract

**Background:**

Hepatitis B virus infection is a significant health problem. Approximately two billion people worldwide have chronic Hepatitis B virus infection and over one million die annually. Hepatitis B virus infection and Human Immunodeficiency Virus co-infection is an emerging concern in the clinical management of patients because of shared routes of transmission.

**Methods:**

Hospital based cross-sectional study was performed from January to June, 2012 at Goba General Hospital. Socio-demographic and possible risk factors data from study subjects were collected using pre-test and structured questionnaire. Venous blood was collected and the serums were tested for Hepatitis B surface antigen and Human Immune Deficiency Virus using commercially available rapid test kits. Data were entered and analyzed using the SPSS software package (version15). Binary and multivariable logistic regressions were used to identify factors associated factors. A p-value of <0.05 was considered statistical significant.

**Result:**

The prevalence of Hepatitis B surface Antigen in this study group was 26 (7.4%). Prevalence of Hepatitis B Human Immune Deficiency Virus Co-infection was about 9 (42.3%) and about 17 (5.1%) of Human Immuno Deficiency Virus negative subjects were positive for Hepatitis B surface Antigen. Risk factors like, hospital admission, multiple sexual partners, HIV status, and unsafe drug injection were found to have significant association with Hepatitis B surface Antigen on binary logistic regression. However, multiple sexual partners and being positive for Human Immuno Deficiency Virus infection were the only significantly associated with Hepatitis B Virus on multivariable logistic regression.

**Conclusion:**

Even though Hepatitis B surface Antigen prevalence is higher among subjects who are Human Immuno Deficiency Virus positive, screening program has to be started in the hospital for all clients regardless of their disease status to prevent the potential spread of the infection.

## Background

Viral hepatitis is a major health problem worldwide and cause acute and/or chronic hepatitis, which can lead to the development of extensive liver scarring (cirrhosis), liver failure, liver cancer and death [1, 2].

Hepatitis B virus is one of the major diseases that causes serious public health problem [3]. World Health Organization estimated that about two billion people worldwide have been infected with HBV, about 350 million people become chronic carriers and over one million people die each year as a result of acute fulminate liver disease or HBV induced cirrhosis and liver cancer [4, 5].

The burden of HBV infection is highest in sub-Saharan Africa [6] and the prevalence of HBV infection in Africa is on average more than 10% [7]. In particular to Ethiopia, Study conducted in Addis Ababa showed that the mean prevalence of HBsAg was 7% [8], at Shashamane General Hospital about 5.7% [9].

Chronically infected hepatitis carriers are able to transmit through contact with their blood, body fluids and secretions. The current treatment for Hepatitis B Virus infection is not curable after the infection progresses to chronic stage and not economical for individuals in developing countries like Ethiopia. HBV is also a major occupational hazard for health workers [10]. Hence, early screening of people who attend hospitals to know the status of these individuals for the infection and identifying associated risk factors are important to undertake effective prevention and control measures [11].

## Methods

### Study design and area

A Hospital based cross-sectional study was conducted at Goba Town located in Bale Zone, South East Ethiopia from January to June 2012. The study participants were individuals in the Outpatient Department of Goba General Hospital during the study period. Goba is a town located in the Bale Zone of the Oromia Region approximately 446 km South West of Addis. This city has a latitude and longitude of 7°0′N 39°59′E and an elevation of 2,743 meters above sea level. In this town there is one general hospital that serves a population of Goba Woreda and surrounding by providing curative and preventive care.

### Sample size determination and sampling techniques

The sample size was calculated using single proportion formula considering the level of confidence 95% (z = 1. 96) and precision of 5% (d =0. 05), since there was no study conducted in the region, the expected estimated prevalence of HBV in the area was set at 50% to yield maximum sample size of 384.

Lottery method was used to recruit subjects for interview and blood sample collection at the Outpatient Department during the study period from January to June 2012.

### Data collection tool and methods

Pre-tested and structured questionnaire which consist of social-demographic information, history of exposure to risk factors in the past years like the history of STI, history of liver diseases, history of invasive procedures like tooth extraction, abortion and ear piercing, history of multiple sexual partner, blood transfusion, history of hospital admission and contact with family having liver diseases. HBV vaccination status of the study subjects also asked during data collection. These data were collected by counselor nurses currently working in the hospital after getting intensive training given to them before the commencement of data collection.

### Specimen collection and processing

After obtaining informed consent, about 5 ml of blood sample was collected by vein-puncture from each of the participants under aseptic conditions from 353 subjects by experienced laboratory personnel working in the hospital and immediately put in a vacutainer tubes containing a clot activator. These tubes were numbered and processed at the time of collection and the assay was performed within one hour of sample collection.

### Serological test

At the site, the HIV status of all study participants was obtained by counsellor nurse using HIV testing algorithm rapid test kit as (KHB, Shanghai Kehua Bio-engineering Co., Ltd. China) for screening and positive samples were re-tested with STAT-PACK. Samples giving discordant results in the two tests were re-examined using tiebreaker, (Uni-Gold HIV, Trinity Biotech PLC, Co. Wick low, Ireland) and the result of this test was considered.

All the serum samples were tested for HBsAg by using LINEAR HBsAg which is quantitative, lateral flow immunoassay was used for detection of HBsAg in serum or plasma. Samples positive for HBV were re-tested for the second time by the same method. Samples repeatedly reactive for HBsAg were considered positive. The subjects positive for HBV were referred and consulting to the internal medicine of the Goba Hospital for further evaluation and treatment.

### Data quality control

Pretest was conducted before actual data collection to ensure the quality of the questionnaire. Trained and experienced laboratory technologist performed laboratory tests. Quality control of both markers was checked based on the manufacturer’s kit instructions. In addition, formation of the coloured band to the control (C) line acts as a procedural control and serves to valid the results.

### Study variables

Independent variables includes social demographic factors and risk factors for HBsAg including history of hospital admission, history of sexually transmitted infections, multiple sexual partners, invasive procedures including tooth extraction, abortion and ear piercing, history of liver disease, contact with people with liver disease, HBV vaccination status and HIV status of the study subjects. The dependent variable was the prevalence of Hepatitis B Surface Antigen.

### Data analysis

The data were cleaned, entered and analysed using computer software (SPSS version 15). The result was summarized using descriptive statistics for social demographic and risk factor variables of Hepatitis B infection. Multiple Logistic regressions were used to identify factors significantly associated with Hepatitis B surface antigen (HBsAg). Finally, p-value of less than 5% was declared as significant association.

### Ethical consideration

The study was conducted after obtaining institutional ethical clearance from Research and Community Service of Madawalabu University. After Separate permission was also obtained from the Goba General Hospital, information about the study was given to all study participants and written informed consent was obtained. The participants were assured that all the information would be kept in utmost confidentiality and the samples would be utilized only for research purpose.

## Results

### Socio demographic characteristics of the study subjects

From the total sample size, 353 were involved making response rate of 91.9%. The majority of the participants 246 (69.7%) were from urban and 203 (57.5%) of them were females. Among the study subjects, 229 (64.9%) were married, 82 (23.2%) were single and 107 (30.3%) of the study subjects belong to 25–34 years of age (Table 1).

**Table 1:** **Socio demographic distribution of study subjects at Goba General Hospital, South East Ethiopia, 2012**

Characteristics		Number	Percentage
Residence	Rural	107	30.3
	Urban	246	69.7
Age	15-24	90	22.5
	25-34	107	30.3
	35-44	63	17.8
	45-54	34	9.6
	54+	59	16.7
Sex	Male	150	42.5
	Female	203	57.5
Marital status	Married	229	64.9
	Single	82	23.2
	Divorced	32	9.1
	Widowed	10	2.8
Religion	Muslim	193	54.7
	Christian	160	45.3
Ethnicity	Oromo	230	65.2
	Amhara	97	27.4
	Tigre	26	7.4
Occupation	Merchant	60	17.0
	House wife	65	18.4
	Student	52	14.7
	Farmer	107	30.3
	Retired	27	7.6
	Governmental employee	38	10.8
	No work	4	1.10
Educational status	Illiterate	85	24.1
	Primary school	153	43.3
	Secondary school	77	21.8
	Diploma and above	38	10.8

### Prevalence’s of HBsAg and distributions of its risk factors

Among 353 study participants tested, HBsAg was detected positive in 26 (7.4%) and 21 found to be positive for HIV infection. Among 21 clients who were positive for HIV, 9 (42.8%) were positive for HBsAg and among 322 tested negative for HIV, 17 (5.1%) of them found to be HBsAg positive respectively.

Regarding History of vaccination, 328 (92.9%) were reported unvaccinated while only 25 (7.1%) reported they were vaccinated for Hepatitis B infection. Of different risk factors considered in this study, history of invasive procedures accounts about 108 (79.4%) followed by history of multiple partner which accounts about 54 (15.3%). Among subjects reported invasive procedure, 108 (78.3%) reported Tooth extraction, 23 (16.7%) Abortion, 7 (5.1%) reported history of ear piercing. Among study subjects screened for HIV infection about 21 (5.9%) were found positive for HIV infection (Table 2).

**Table 2:** **Prevalence of hepatitis b virus and its risk factors among study subjects at Goba General Hospital, South East Ethiopia, 2012**

*Variables*		*Frequency (n = 353)*	Percentage
HBV infection status	No	327	92.6
	Yes	26	7.4
History of Liver diseases	No	306	86.7
	Yes	47	13.3
History of contact with Liver diseases in family	No	310	87.8
	Yes	43	12.2
History of STI	No	310	87.8
	Yes	43	12.2
History of invasive Procedures	No	217	61.5
	Yes	136	38.5
Unsafe drug injection	No	335	94.9
	Yes	18	5.1
Multiple partner	No	299	84.7
	Yes	54	15.3
Blood transfusion	No	335	94.9
	Yes	18	5.1
HIV status	No	332	94.1
	Yes	21	5.9
History of hospital admission	No	269	76.2
	Yes	84	23.8
History of HBV vaccination	Unvaccinated	328	92.9
	Vaccinated	25	7.1

Regarding the history of vaccination, about 328 (92.9%) were reported unvaccinated while only 25 (7.1%) reported that they were vaccinated for Hepatitis B infection. Of different risk factors considered in this study, history of invasive procedures accounts about 108 (79.4%) followed by history of multiple partner which accounts about 54 (15.3%). Among the subjects reported invasive procedure; 108 (78.3%) reported Tooth extraction, 23 (16.7%) Abortion, 7 (5.1%) reported history of ear piercing. Among study subjects screened for HIV infection, about 21 (5.9%) were found positive for HIV infection.

In this study, significantly high prevalence of HBsAg marker was observed among individuals who have history of hospital admission and Seropositive for HIV infection. Among 21 HIV positive individuals, 9 (42.8%) were also positive for Hepatitis B infection. The statistical association between the distribution of HBsAg and HIV indicates a significant association (*x*^2^: 41.2; OR; 13.8; p; 0.000).

Risk factors like, Hospital admission, multiple sexual partnerships, HIV Infection and unsafe drug injection was found to have significant association with hepatitis B infection on binary logistic regression while HIV infection status and History of multiple sexual partner found significantly associated with Hepatitis B virus infection on multivariable analysis (Table 3).

**Table 3:** **Results from multiple logistic regressions on factors associated with hepatitis b surface antigen among study subjects at Goba General Hospital, South East Ethiopia, 2012**

Risk factors		COR	P	95% CI	P	AOR	95% CI
Hepatitis immunization	No	-	0.998	-	0.998	-	-
	Yes	Reference group					
History of hospital admission	No	3.03	0.008	1.35-6.85	0.214	0.506	0.17-1.48
	Yes	Reference group					
History of blood transfusion	No	1.62	0.54	0.35-7.46	0.322	2.574	0.39-16.72
	Yes	Reference group					
History of unsafe injection	No	4.0	0.021	1.23-13.39	0.894	1.119	0.21-5.89
	Yes	Reference group					
Multiple partners	No	4.0	0.021	1.23-13.39	0.001	0.116	0.03-0.39
	Yes	Reference group					
History of STI	No	1.7	0.260	0.65-5.01	0.588	1.521	0.33-6.93
	Yes	Reference group					
Invasive procedure	No	1.9	0.100	0.88-4.34	0.094	0.429	0.16-1.15
	Yes	Reference group					
Liver Diseases	No	1.6	0.360	0.58-4.52	0.713	0.498	0.01-20.53
	Yes	Reference group					
Contact with family having Liver diseases	No	1.8	0.260	0.64-5.10	0.661	2.316	0.05-98.47
	Yes	Reference group					
HIV infection	No	0.07	0.000	0.03-0.19	0.000	0.068	0.02-0.23
	Yes	Reference group					

AOR: Adjusted Odds Ratio, CI: Confidence Interval, COR: Crude Odds Ratio, P: p- Value.

## Discussions

In Ethiopia, data about HBV are scarce, particularly absent in selected hospital. Hence, the present study tried to determine the seroprevalence of HBV and associated factors among individuals attending Goba General Hospital for seeking care or other services during the study period.

In this study, the overall prevalence the rate of hepatitis B virus infection among individuals visiting Goba General Hospital was 7.4% which is higher than finding reported by *Abebe et al.* for the general population of Addis Ababa which was about 6.1% [8]. This difference might be due to the difference in study participants and sample size.

In this study, higher HBsAg 9 (42.8%) positivity rate was observed among HIV positive individuals. This is comparable with studies done by Burnett *et al.* indicating an increased occurrence of HBV among HIV positive individuals [12]. This could be due to the fact that both HBV and HIV share common means of transmission and risk factors and weakened immune systems. However, in contrast to this study, a study done in Addis Ababa [13] and study from South Africa [14] shows the low prevalence rate of HBV among HIV positive individuals. This might be partly due to effect that some HIV drugs such as lamivudine had on HBV and in turn, an elimination of HBsAg [15]. However, this could not be the entire explanation as HBsAg was still observed in relatively lower rate among study subjects naïve for ART [13].

Having a history of multiple sexual partners also found to be significant with Hepatitis B infection. This is because mostly this infection occurs among high risk populations that include injection drug users, person with multiple heterosexual patterns [12]. Identified risk factors like blood transfusion; tooth extraction was not associated with the infection in our study. This indicates that health care workers in the selected set up strictly use aseptic techniques which really help in prevention of the infection.

## Conclusion

Even though Hepatitis B surface Antigen prevalence is higher among subjects who are Human Immuno Deficiency Virus positive, screening program has to be started in all health facilities for all clients regardless of their sero-status to prevent the potential spread of infection.

### Recommendations

Health care workers should use personal protective devices while giving a care for the patients regardless of their disease status to prevent the potential spread of this viral infection.

Woreda Health Bureaus and other relevant stakeholders should work on integrating screening program for HBV infection at the health services like Goba Hospital to prevent the infection.

Nationwide studies should be conducted to determine the magnitude of HBV and its risk factors to get enough information that help for planning policy to take preventive measures.
